# The Protein Kinase CK2 Mediates Cross-Talk between Auxin- and Salicylic Acid-Signaling Pathways in the Regulation of *PINOID* Transcription

**DOI:** 10.1371/journal.pone.0157168

**Published:** 2016-06-08

**Authors:** Laia Armengot, Eleonora Caldarella, Maria Mar Marquès-Bueno, M. Carmen Martínez

**Affiliations:** Departament de Bioquímica i Biologia Molecular, Universitat Autònoma de Barcelona, Bellaterra (Barcelona), Spain; Iwate University, JAPAN

## Abstract

The protein kinase CK2 is a ubiquitous and highly conserved enzyme, the activity of which is vital for eukaryotic cells. We recently demonstrated that CK2 modulates salicylic acid (SA) homeostasis in *Arabidopsis thaliana*, and that functional interplay between CK2 and SA sustains transcriptional expression of *PIN-FORMED* (*PIN*) genes. In this work, we show that CK2 also plays a key role in the transcriptional regulation of *PINOID* (*PID*), an AGC protein kinase that modulates the apical/basal localization of auxin-efflux transporters. We show that *PID* transcription is up-regulated by auxin and by SA and that CK2 is involved in both pathways. On the one hand, CK2 activity is required for proteosome-dependent degradation of AXR3, a member of the AUX/IAA family of auxin transcriptional repressors that must be degraded to activate auxin-responsive gene expression. On the other hand, the role of CK2 in SA homeostasis and, indirectly, in SA-driven *PID* transcription, was confirmed by using Arabidopsis *NahG* transgenic plants, which cannot accumulate SA. In conclusion, our results evidence a role for CK2 as a functional link in the negative cross-talk between auxin- and SA-signaling.

## Introduction

The phytohormone auxin plays a central role in plant development and in the plant’s responses to environmental stimuli such as gravitropism, phototropism and root plastic growth [1]. An important finding in the last decade was that auxin is not uniformly distributed within the plant tissues and that establishment of auxin maximum and minimum is a key step of organogenesis [2,3]. The asymmetric distribution of indoleacetic acid (IAA), the most abundant auxin in plants, relies mainly on cell-to-cell transport, also called polar auxin transport (PAT) [3]. Several auxin carriers located at the plasma membrane have been identified to date. The AUX1/LAX proteins are auxin-influx transporters that facilitate the entry of apoplastic IAA into the cell. In addition, two families of proteins acting as auxin-efflux transporters have been reported: 1) the PIN-formed (PIN) proteins, and 2) some members of the multi-drug-resistant/P-glycoprotein (MDR/PGP) subfamily of the ATP-binding cassette (ABC) proteins [4–9]. The plasma membrane-resident PIN proteins are the most studied regulators of PAT. They show polar distribution at the plasma membrane, which is dependent on the cell type [10]. This highly specific targeting of the PIN proteins is an intriguing question, and it is thought that signals derived from cell membrane composition (in particular sterols) [11], post-translational protein modification, and cell-type specificity might interplay in its regulation [12–15]. PIN phosphorylation by the protein kinase PINOID (PID) was one of the first described regulatory signals involved in PIN polar localization [16,17]. PID is a plasma-membrane (PM)-associated kinase that directs targeting of PIN proteins to the apical side of the PM by phosphorylating these transporters in their large central hydrophilic loop [18,19]. PID belongs to the family of AGC protein kinases [20]. It has been reported that two other members of this family, WAG1, and WAG2, which are the closest homologues to PID [21], also instruct recruitment of PINs into the apical recycling pathway by phosphorylating the middle serine in three conserved TPRXS(N/S) motifs within the PIN central hydrophilic loop [14]. Moreover, protein kinase D6PK, also belonging to the AGCVIII family, phosphorylates particular members of the PIN protein family and also contributes to the regulation of auxin transport [22]. More recently, it was shown that the ABCB/PGP family of auxin-efflux carriers were also substrates of PID [23].

Auxin acts in concert with other phytohormones, such as cytokinins, gibberellins and ethylene, to fine-tune specific developmental programs or to respond to environmental factors [24–26]. Importantly, antagonistic action between auxin and salicylic acid (SA) was recently reported, which is exploited in plant defense. Some pathogens have evolved the ability to produce auxin or stimulate auxin biosynthesis in plant host cells to disrupt normal growth of infected plants [24,27]. Plants, on their side, evolved mechanisms to reduce auxin signaling upon pathogen infection by producing elevated amounts of SA, which causes transcriptional repression of genes encoding auxin receptors (proteins TIR1 and AFB1) [28] and of genes encoding some members of the auxin transporters family [29].

Protein kinase CK2 is a ubiquitous and constitutively active protein kinase, the activity of which is essential for cell survival in all eukaryotes [30,31]. We recently reported that inhibition of CK2 activity in Arabidopsis plants had important effects on auxin signaling, particularly on PAT [32] and that *PID* was highly overexpressed in roots of CK2-defective plants. In addition, many members of the PIN protein family were misregulated and also mislocalized [32]. As PID plays a prominent role in the regulation of PIN localization and consequently on PAT, we wanted to study further the molecular signals that regulate *PID* transcription and the role of CK2 in this regulation. We had previously demonstrated that *PID* is transcriptionally up-regulated by SA [29] and in this work we provide new data regarding the molecular mechanisms involved in *PID* transcriptional regulation. We propose that CK2 has a role in mediating the SA and auxin cross-talk that regulates *PID* transcription.

## Materials and Methods

### Plant material

*Arabidopsis thaliana* (Col-0 ecotype) and the transgenic lines used in this work were grown at 21 to 22°C under a 16 h photoperiod light (140 μE m^-2^ sec^-1^). For *in vitro* germination and culture, seeds were surface sterilized and grown in Murashige and Skoog (MS) plates (Duchefa Biochemie BV, http://www.duchefa.com/) supplemented with 0.5% (w/v) sucrose and 1.2% (w/v) agar. Generation of dominant-negative CK2 mutant plants (henceforth named CK2mut plants) was described in [31]. Arabidopsis *NahG* transgenic plants [33] were obtained from P. Tornero (IBMCP-Valencia, Spain). The Arabidopsis auxin-signaling mutants *tir1* and *nph4/arf19* were purchased from The European Arabidopsis Stock Centre (NASC). Arabidopsis seedlings containing the *HS*::*AXR3*.*3NT-GUS* construct were a kind gift of Mark Estelle (Salk Institute, La Jolla, CA, USA).

### Plant treatments

Expression of *CK2mut* transgene was induced with 1 μM dexamethasone (Dex) (Duchefa, www.duchefa.com) for 48 h, and controls with Dex solvent (ethanol) were performed in all cases. Salicylic acid (SA) was dissolved in ethanol and treatments were performed at 0.25 mM for 4 h. The final concentrations and solvents for other drugs used in this study were the following, unless otherwise indicated: 25 μM 4,5,6,7-tetrabromobenzotriazol (TBB) (Calbiochem, www.emdmillipore.com) (dimethyl sulfoxide, DMSO), 50 μM MG132 (Sigma-Aldrich,) (DMSO), 10 mg/L cycloheximide (Sigma) (ethanol), 10 μM 1-naphthaleneacetic acid (NAA) (Sigma) (ethanol) and 1 μM 2,4-dichlorophenoxyacetic acid (2,4-D) (Sigma) (ethanol). All these treatments were performed in liquid MS medium for 4 h (unless otherwise indicated) and appropriate controls with the corresponding solvents were always carried out.

### Heat induction and GUS assays

Seedlings were grown in MS plates and then transferred to liquid medium before the treatments. Seven-days-old *HS*::*AXR3*.*3-GUS* seedlings were heat shocked (HS) for 2 h at 37°C and then left for one additional hour at room temperature with shaking. Treatments with SA (0.25 mM in ethanol) were carried out for 3 h, starting at the same time as HS. Treatments with TBB (25 μM in DMSO) and MG132 (50 μM in DMSO) (Sigma) were carried out for two hours, starting one hour after launching HS. Treatment with 2,4-D (5 μM in ethanol) was carried out for one hour, starting at the end of HS. GUS staining in Arabidopsis roots was performed as in [34]; seedlings were mounted with 50% glycerol (v/v) and observed with a Leica DMRB microscope (Leica, http://www.leica.com). Images were taken with a Leica DFC500 digital camera. Fluorimetric assays of GUS activity were carried out with 4-methylumbelliferyl-β-D-glucuronide (MUG) (Sigma) as a GUS substrate, following the procedure described in [35]. Plants were sampled at 20 min intervals and stored in liquid nitrogen until GUS activity was measured. Protein extracts were obtained in GUS buffer (50mM KPO_4_, pH 7.0, 0.1% sarkosyl, 0.1% Triton X-100, 10mM β-mercaptoethanol, 10mM EDTA). Equal volumes of protein extracts and of MUG solution (2mM in GUS buffer) were incubated for 16 h in darkness. The reaction was stopped with 900 μl of 0.2M Na_2_CO_3_ and fluorescence was measured in a spectrophotometer Cary Eclipse (Varin) with λ_excitation_ at 365 nm and λ_emission_ at 450 nm. Extracts were prepared from 10 seedlings and data were normalized against total protein content determined by Bradford assays. Three biological replicates for each sample were performed.

### Quantitative RT-PCR analysis

Total RNA was extracted with Trizol (Life Technologies, http://www.lifetechnologies.com/) and first-strand cDNA was synthesized using the iScript cDNA synthesis kit (Bio-Rad Laboratories). Quantitative PCR was performed using a Bio-Rad CFX96 real-time PCR Detection System and SYBR Green Master Mix (Bio-Rad Laboratories). The specificity of the PCR reactions was confirmed by melting curve analysis (55–95°C). The -ΔCt values were calculated relative to either *EF-1-α* or *18S* [36]. The specific primers and annealing temperatures for *EF-1- α*, *PIN* and *PID* genes were previously described [29,32]. The specific primers for *18S* were GATGAGCCTGCGTAGTATTAG (forward) and AGTCATTCCGAAGAACACTTGC (reverse). Statistical analyses of data were performed either with Student’s *t*-test (P ≤ 0.05) or with ANOVA (P ≤ 0.05) and post hoc pairwise comparisons with Tukey’s test (Excel, Microsoft).

## Results

### Expression of *PID* in CK2-defective plants and gene transcriptional responses to auxin

**Fig 1:**
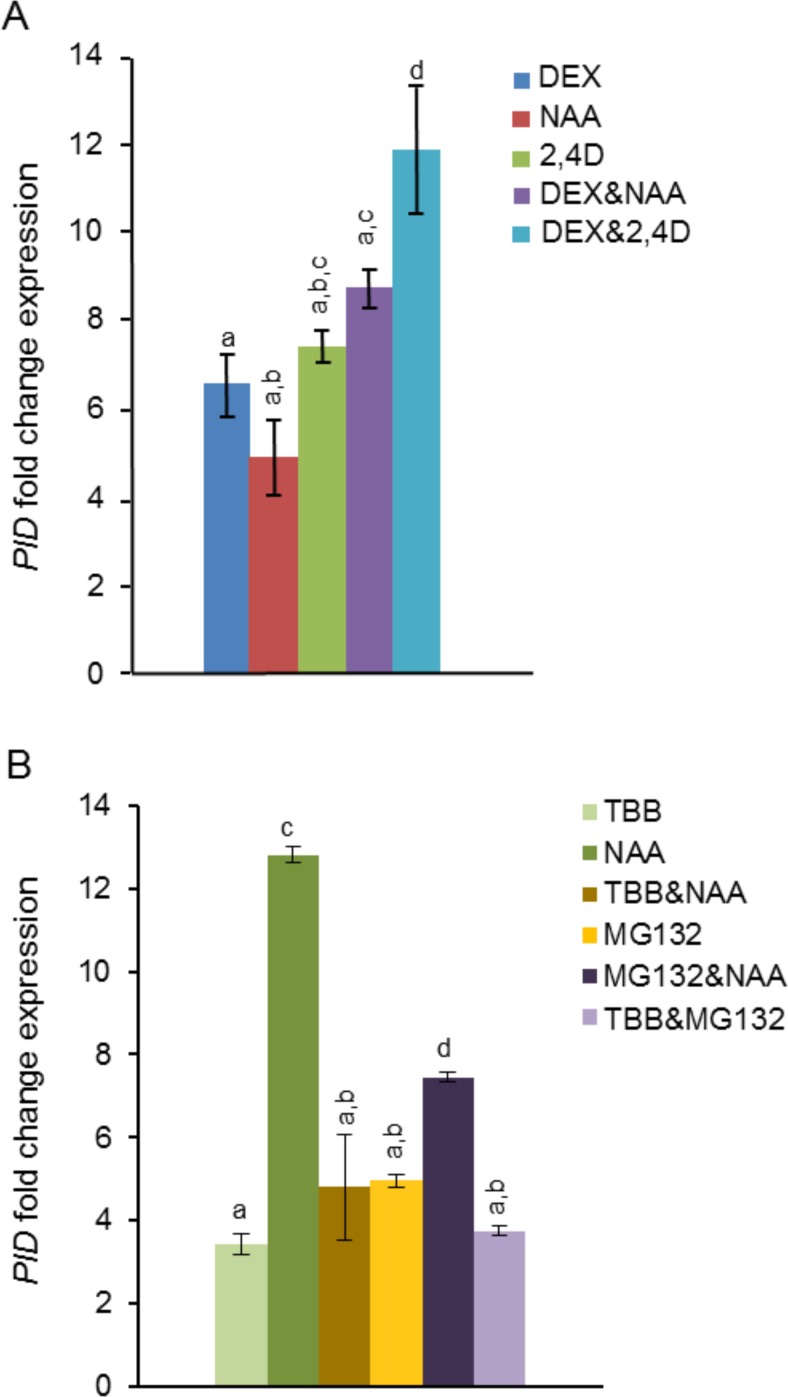
*PID* transcriptional regulation by auxin in wild-type and CK2-defective roots. (A) *PID* transcriptional responses to auxin in CK2mut plants. Transcript levels were measured in roots and normalized to those of *EF-1-α* gene. (B) *PID* transcriptional responses to auxin in TBB-treated plants and interplay with proteosome activity. Data were expressed as fold changes of gene expression relative to the levels measured in control plants. Graphs show the mean of three biological replicates ± standard deviation. Same letters above the bars indicate no significant differences from each other (ANOVA P≤0.05). Dex, dexamethasone; NAA, 1-naphthaleneacetic acid; 2,4-D, 2,4 dichlorophenoxyacetic acid; TBB, 4,5,6,7-tetrabromo benzotriazol; *PID*, *PINOID*. The symbol & indicates simultaneous treatments.

We have previously demonstrated that a dexamethasone (Dex)-inducible dominant-negative allele of CK2 (*CK2mut*) could be successfully used to deplete CK2 activity in Arabidopsis plants [31,32]. Moreover, Dex-induced CK2mut plants exhibited strong up-regulation of *PINOID* (*PID*) transcription [32]. As PID is a key regulator of polar auxin transport (PAT), a process in which CK2mut plants are defective, we became interested in studying the interplay between CK2 activity and the signaling pathways controlling *PID* transcription. We first studied *PID* transcriptional responses to auxin both in wild-type and CK2mut backgrounds. All our experiments were performed in roots to discard possible effects due to tissue-specific factors. Fig 1A shows that *PID* was up-regulated by auxin, using either NAA or 2,4-D. The effect was stronger with 2,4-D, which might be a consequence of the slower metabolic rate of 2,4-D and the different transport mechanisms of those two auxin analogs [37]. Moreover, auxin-driven *PID* up-regulation was potentiated in the CK2mut background, being more significant with 2,4-D. These results might suggest that CK2 is involved in the regulation of auxin signaling or that an additional pathway, becoming more active after inhibition of CK2, regulates *PID* transcription. Moreover, both hypotheses are not mutually exclusive.

It has been reported that the AGC protein kinases PID, WAG1 and WAG2 are the main regulators of the apical recycling pathway that control PIN localization [14,38]. We investigated whether *WAG1* and *WAG2* genes showed the same pattern of transcriptional regulation as *PID*. Our results show that *WAG1* was overexpressed in Dex-induced CK2mut plants, whereas WAG2 was underexpressed under the same conditions (S1 Fig). Moreover, *WAG1* did not show significant transcriptional changes in response to auxin, whereas *WAG2* was slightly down-regulated under the same conditions. So, at least in roots, *PID*, *WAG1* and *WAG2* exhibit differential transcriptional regulation, which might reflect tissue-specific specialization of these three kinases, as has been suggested by other authors [14], and/or the existence of feed-back mechanisms to secure enough levels of functionally redundant proteins with compensatory properties.

To further investigate the interplay between auxin and CK2, we also measured the transcriptional responses of *PIN-FORMED (PIN)* genes and of *AUX1* gene to auxin. Under our experimental conditions, *PIN1*, *PIN7* and AUX1 were significantly induced both by NAA and 2,4-D, whereas *PIN2* and *PIN4* were unresponsive and *PIN3* was marginally induced only by 2,4-D (S2 Fig). Moreover, CK2 inhibition and NAA/2,4-D treatments had synergistic effects on *PIN7* transcriptional activation. To the contrary, *PIN1*, *PIN3* and *AUX1* responses to 2,4-D were impaired in the CK2mut background. These results corroborate the major impact of CK2 activity in the auxin-driven transcriptional regulation of auxin-efflux transporters, and also reveal a large variability in the gene responses. Other authors showed that *PIN* expression was controlled by auxin in a tissue- and cell-type-specific manner, and that inhibition of auxin transport with NPA produced transcriptional changes that were concordant with cross-regulation of *PIN* gene expression [39]. Our results are in agreement with those of [39], as we detected differential auxin-induced *PIN* transcriptional responses under conditions of impaired auxin transport. However, as our study was carried out in whole roots, the cell-type specific effects could not be measured. Moreover, we have already reported that the *PIN* gene family was differentially regulated by SA in Arabidopsis roots [29]. Thus, the combined treatments of Dex (producing increased SA levels and impaired auxin transport) and auxin in CK2mut roots, was likely to produce differential transcriptional responses within the different members of the *PIN* gene family.

### Interplay between CK2 and auxin in the regulation of *PID* transcription

Auxin-driven gene regulation involves transcriptional and post-transcriptional changes that can occur very rapidly (within minutes). Moreover, CK2 is a pleiotropic protein kinase involved in many basic biological functions, such as gene transcription, protein translation, DNA repair or cell cycle control [40,41]. This raised the possibility that our long-term inductions of the *CK2mut* transgene (48 h of Dex treatments) were affecting too many processes, hampering detection of early and transient auxin effects. To circumvent this possibility and achieve a rapid inhibition of CK2 activity *in planta*, we used the specific CK2 inhibitor 4,5,6,7-tetrabromo benzotriazol (TBB) [42]. We have previously shown that TBB inhibited CK2 activity rapidly, it did not interfere with the activity of other Arabidopsis kinases, and induced accumulation of *PID* transcripts more rapidly than Dex-treatments of CK2mut plants [32,43]. In this work we show that incubation with 25 μM TBB for 4 h increased *PID* transcript levels in WT roots (Fig 1B). Moreover, simultaneous treatment with TBB and NAA impaired the NAA-induced *PID* up-regulation, contrary to the effect observed in Dex-treated CK2mut plants, suggesting that CK2 activity is required for the initial steps of auxin-induced *PID* transcription. Surprisingly, incubation with the proteosome inhibitor MG132 promoted accumulation of *PID* transcripts, and only partially impaired the *PID* transcriptional response to NAA (compare MG132, MG132&NAA, and NAA data, Fig 1B). Moreover, incubation with TBB, MG132 or TBB&MG132 had the same effects on *PID* transcription, indicating that both drugs were acting through the same pathway. As auxin-induced gene transcription is normally blocked in the presence of proteosome inhibitors (due to stabilization of Aux/IAAs [35]), these data unveiled the existence of additional stimuli other than auxin controlling *PID* transcription.

**Fig 2:**
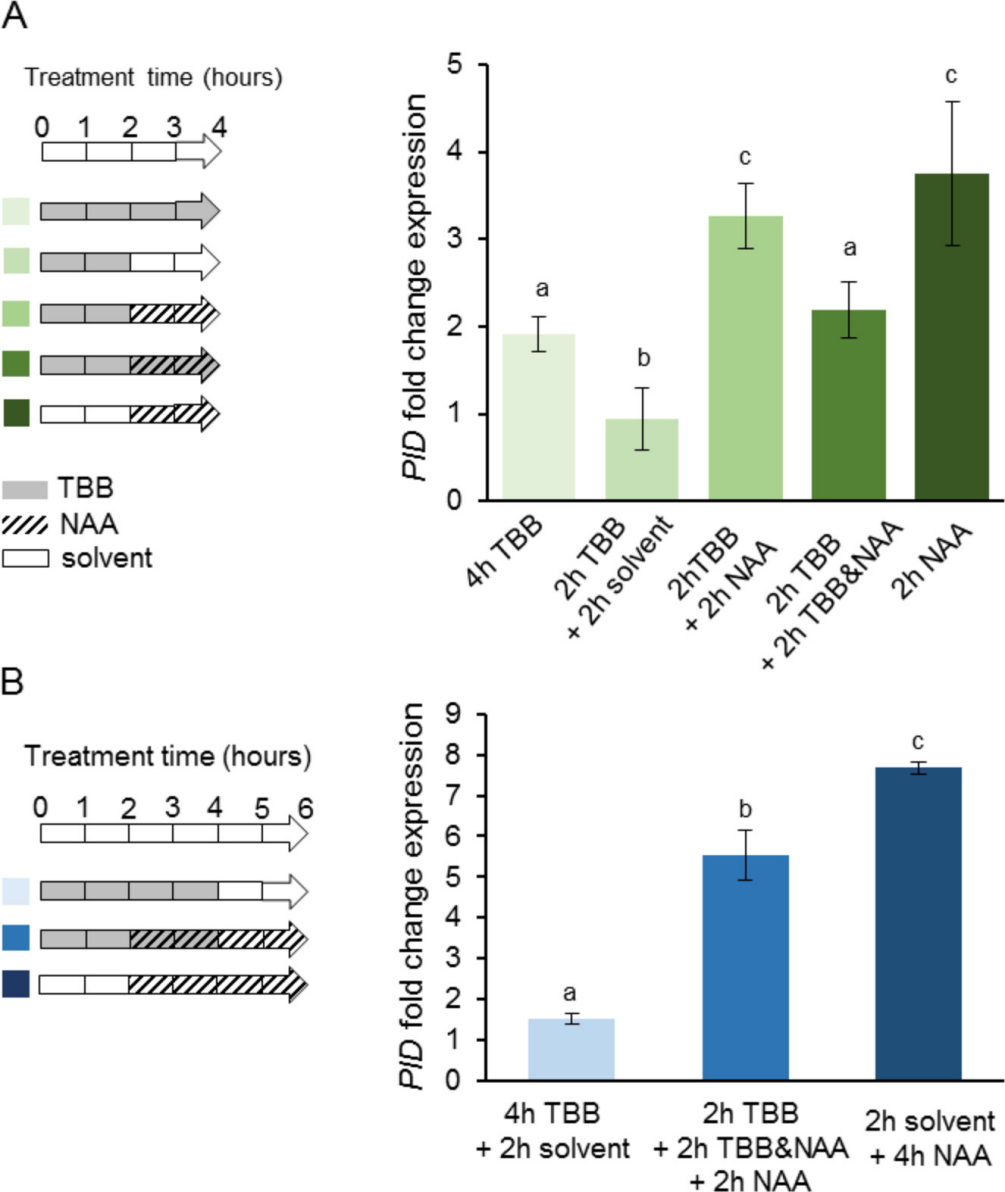
Interplay between CK2 and auxin in the regulation of *PID* transcription. *PID* transcriptional responses to auxin were measured in Arabidopsis roots pre-treated with TBB for 2 h and then submitted to different combinations of TBB and NAA treatments, as indicated. *PID* transcript levels were measured after 4 h (A) and after 6 h (B). Data were obtained and represented as in Fig 1. Statistical analyses were carried out and represented as in Fig 1. TBB, 4,5,6,7-tetrabromo benzotriazol; NAA, 1-naphthaleneacetic acid. The symbol & indicates simultaneous treatments and the symbol + indicates consecutive treatments.

We then further analyzed the auxin mediated *PID* up-regulation following TBB pre-treatments. The experimental design is described in Fig 2A and 2B. Our results show that auxin addition after TBB removal (2 h TBB + 2 h NAA) induced *PID* transcription to similar levels as those obtained by incubation with NAA (2 h NAA) (Fig 2A). To the contrary, TBB inhibited the auxin response when both compounds were simultaneously used (2 h TBB + 2 h TBB&NAA). Moreover, when TBB was removed after the co-treatment (2 h TBB + 2 h TBB&NAA + 2 h NAA) *PID* transcript levels increased, reaching levels close to those of the NAA treatment (4 h NAA) (Fig 2B). These results corroborated the idea that CK2 activity is required early in auxin-induced *PID* transcription.

**Fig 3:**
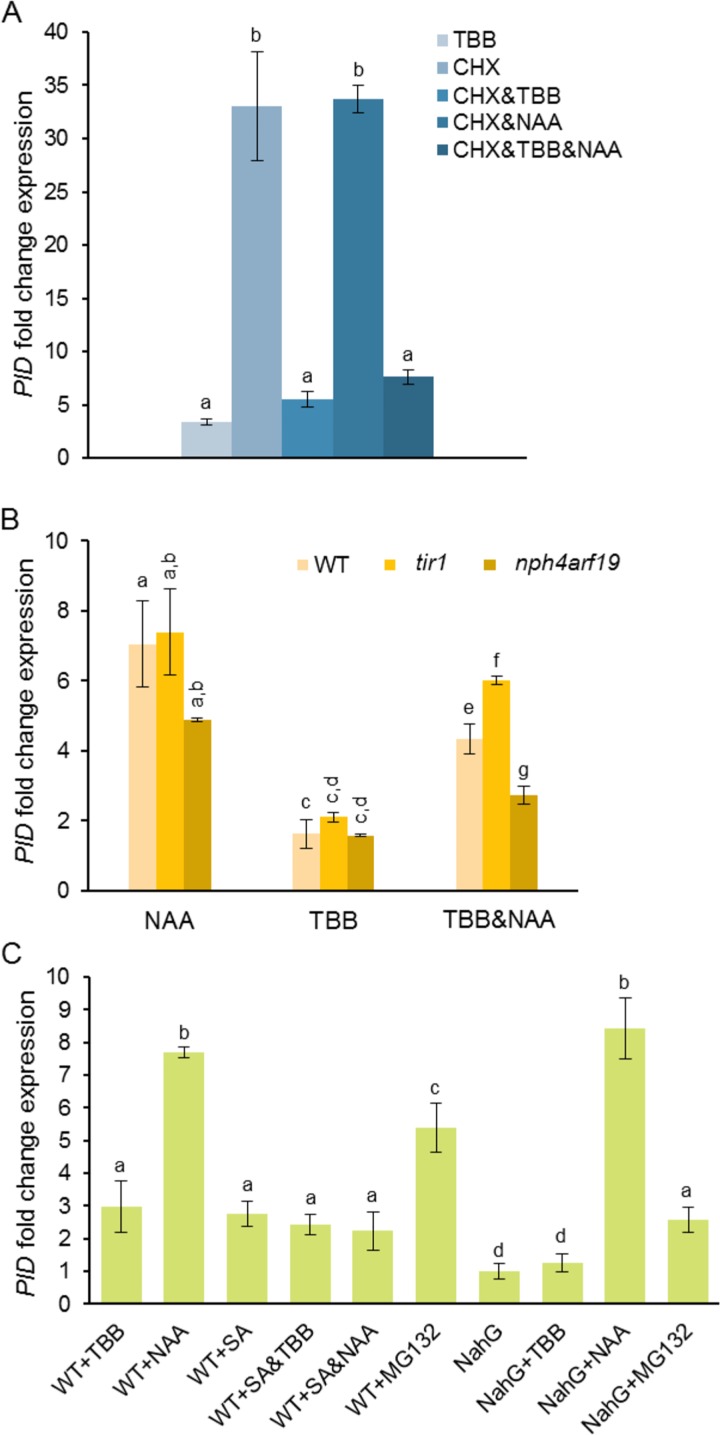
Pathways that regulate *PID* transcription. (A) Interplay between CK2 and *de novo* protein synthesis in modulating *PID* transcriptional responses. Transcript levels were normalized to those of *18S* gene. (B) *PID* transcriptional responses to NAA in auxin-signaling mutants. Transcript levels were normalized to those of *EF-1-α* gene. (C) *PID* transcriptional responses to SA in WT and SA-defective roots (*NahG*). Transcript levels were normalized to those of *EF-1-α* gene. Data were obtained and represented as in Fig 1. Statistical analyses were carried out and represented as in Fig 1. TBB, 4,5,6,7-tetrabromo benzotriazol; CHX, cycloheximide; NAA, 1-naphthaleneacetic acid. *nph4*, nonphototropic hypocotyl 4; *arf19*, auxin-responsive factor 19; *tir1*, transport inhibitor response 1; *NahG*, salicylate hydroxylase. The symbol & indicates simultaneous treatments.

To get deeper insights about the transcriptional regulation of *PID* by auxin, we measured *PID* expression in the presence of cycloheximide (CHX) (an inhibitor of protein synthesis) (Fig 3A), and in Arabidopsis auxin-signaling mutants (Fig 3B). CHX produced high accumulation of *PID* transcripts, confirming that *PID* expression is controlled by unstable short-lived transcriptional repressors (Fig 3A). Moreover, *PID* transcript levels were similar in CHX and CHX&NAA roots, indicating that *de novo* synthesis of any factors is not required for the auxin response and that *PID* is a primary response gene to auxin. The effect of cycloheximide was impaired in the presence of TBB, suggesting that inhibition of CK2 either stabilizes auxin repressors and/or that CK2 is necessary for the activity of auxin responsive factors. Incubation with NAA could not reverse the inhibitory effect of TBB&CHX, supporting the idea that CK2 activity is required for targeting the auxin repressors for degradation. As a control, we also measured the effects of CHX on *PIN* transcript levels (S3 Fig), showing that CHX produced accumulation of transcripts (which agrees with the results shown in [39]) and that TBB significantly impaired the CHX effects. The exception was *PIN4* that, as is shown in S2 Fig, was unresponsive to auxin under the conditions used in this work.

We also analyzed *PID* expression in Arabidopsis *tir1* and *nph4arf19* signaling mutants. *tir1* is a loss-of-function mutant of one of the members of the TIR/AFB family of auxin receptors with overlapping functions [44]. *nph4arf19* is a double loss-of-function mutant of two locus encoding auxin response factors (NPH4 and ARF19). NPH4 has been proposed to be involved in the regulation of all the auxin-inducible genes and ARF19 is a transcriptional activator of early auxin responses [45,46]. Fig 3B shows that the *PID* response to NAA is independent of TIR1, indicating that other members of the receptor family must be involved [44]. Moreover, the auxin-induced *PID* overexpression was slightly reduced in the *nph4arf19* mutant, suggesting that NPH4 and/or ARF19 are involved in *PID* transcription and that additional ARFs might be necessary as well. The effect of the *nph4arf19* mutation was more clearly observed upon inhibition of CK2 activity (TBB&NAA), as TBB treatments produced stronger repression of NAA-induced *PID* transcription in *nph4arf19* than in WT plants.

### Involvement of CK2 in the SA-regulated *PID* transcription

We recently reported that *PID* transcription was up-regulated by SA and that CK2mut plants and TBB-treated plants contained very high levels of SA [29]. To further explore the contribution of SA to *PID* transcriptional regulation, we used *NahG* plants that are SA-defective due to overexpression of a bacterial salicylate hydroxylase [33]. We previously demonstrated that TBB-treated *NahG* roots were unable to accumulate SA [29] and we show here that *PID* transcript levels in *NahG* and *NahG*+TBB roots were similar to those in WT (Fig 3C). These results suggest that accumulation of SA is required for the up-regulation of *PID* transcription in CK2-defective plants. Moreover, results in Fig 3C show that exogenous SA damps down the NAA-induced *PID* transcriptional activation (compare WT+NAA, WT+SA&NAA and *NahG*+NAA data), which is in agreement with the reported repression of auxin-signaling by SA [28]. In addition, incubation of TBB-treated roots with exogenous SA cannot induce *PID* transcription further (same transcript levels were obtained in WT+TBB and WT+SA&TBB roots), suggesting that SA levels necessary to induce *PID* transcription reached saturation in TBB-treated roots Surprisingly, *PID* transcripts increased in MG132-treated *NahG* plants, although to a lower extent than in MG132-treated WT plants. As we will discuss later, the protein levels of some positive regulators of the SA-signaling pathway are controlled by rapid turnover, in which the 26S proteosome activity is functionally involved. Thus, inhibition of proteosome might lead to a constitutive activation of the SA-signaling pathway.

### Inhibition of CK2 activity increases the stability of the AXR3/IAA17 auxin repressor

**Fig 4:**
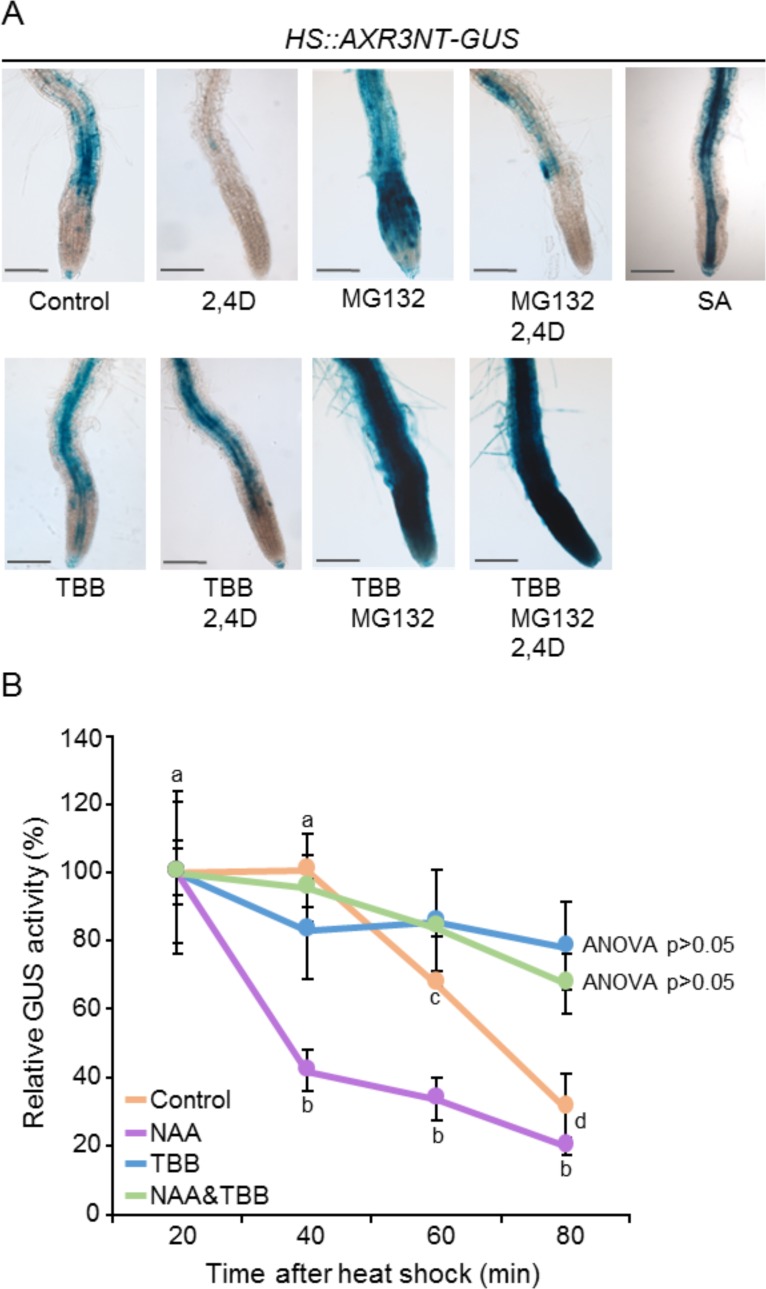
CK2 activity regulates the stability of AXR3, an auxin transcriptional repressor. (A) GUS activity staining. Seven-day-old *HS*::*AXR3*.*3-GUS* seedlings were heat shocked for 2 h (control) and treated with 2,4-D, MG132, SA and TBB in different combinations, as indicated. n = 10–20. (B) Fluorimetric assay of GUS activity. Relative activity is expressed as percentage of the 20-min GUS activity levels. Error bars indicate standard deviations. Same letters above the bars indicate not significant differences from each other (ANOVA P≤0.05). Abbreviations: 2,4-D, 2,4 dichlorophenoxyacetic acid; TBB, 4,5,6,7-tetrabromo benzotriazol.

It has been previously shown that SA causes global repression of auxin-related genes, including *TIR1* that encodes a member of the AFB family of auxin receptors [28]. The same authors demonstrated that SA stabilizes the auxin repressor AXR3, resulting in inhibition of auxin responses. Thus, we wondered if the same was true in CK2 defective plants, which contain high levels of endogenous SA. For this purpose, we analyzed the transcript profiles of Dex-induced CK2mut plants, previously reported by our group [41] and publicly available at NASCARRAYS-642 (http://affymetrix.arabidopsis.info/). Slight repression of *TIR1* but not of other members of the AFB family was found, suggesting that auxin perception was not impaired in these plants. We then studied the stability of the auxin repressor AXR3/IAA17. We used *HS*::*AXR3NT-GUS* Arabidopsis seedlings, in which the amino-terminal domains I and II of AXR3 (AXR3NT) (controlling protein ubiquitination and degradation) were fused to GUS, and the expression of the chimeric protein was placed under the control of the soybean heat-shock (HS) promoter [35]. Fig 4A shows that GUS activity was detected in Arabidopsis roots 2 h after HS treatment (control) and disappeared when the HS treatment was followed by incubation with 2,4-D. Moreover, as expected, the 2,4-D-induced AXR3 degradation was prevented in the presence of the proteosome inhibitor MG132. These results confirmed that AXR3-GUS chimeric protein was post-translationally regulated in a similar way than wild-type AXR3, i.e. by auxin-induced degradation via the proteosome. 3 4A also shows that incubation with SA or TBB increased AXR3 stability. Furthermore, exogenous 2,4-D added to TBB-treated plants was unable to target AXR3-GUS for degradation, as GUS activity did not decrease. Moreover, simultaneous incubation with MG132 and TBB potentiated the MG132 effect, suggesting that TBB and MG132 are acting on different steps of the degradation pathway (Fig 4A).

Finally, we quantified the effects of TBB and auxin on AXR3 stability by using a fluorimetric assay. AXR3NT-GUS activity was measured at 20-min intervals after heat shock using 4-methylumbelliferyl-β-D-glucuronide (MUG) as a GUS substrate (Fig 4B). Whereas AXR3NT-GUS levels decreased rapidly in control plants and NAA-treated plants, GUS activity remained high in TBB-treated roots (blue line, ANOVA>0.05), with only a slight but not significant decrease at 80 min after HS (to 68% of the initial GUS activity value). Moreover, treatment with exogenous NAA was unable to reverse the TBB effect. We conclude that protein kinase CK2 functions as a regulator of AXR3 stability, which is in agreement with our previous hypothesis that CK2 activity was required in the early steps of auxin-driven gene transcription.

## Discussion

We have previously reported that inhibition of protein kinase CK2 in Arabidopsis seedlings resulted in accumulation of high levels of *PINOID* transcripts [32]. As protein kinase PINOID is a key regulator of polar auxin transport [13,15,23,47], we wanted to increase our knowledge about the role of CK2 on the hormonal control of *PID* transcription. Moreover, very little is known about the signaling pathways in which plant’s CK2 participates and, thus, this study might contribute to deeper insights. We used both genetic and pharmacological tools to inhibit CK2 activity, because these two approaches gave us complementary information. CK2 inhibition in CK2mut plants was achieved by the overexpression of a CK2α catalytically inactive subunit that accumulates after 24 to 48 h of Dex-treatments, producing a dominant-negative effect [31]. This long lapse of time until CK2 inhibition was complete, together with the ubiquitous nature of CK2, made it difficult to unravel primary from secondary effects of CK2 inhibition. To the contrary, as mentioned above, TBB is a specific inhibitor of CK2 activity [43,48], the effects of which can be measured after few hours. We took advantage of this approach to study the auxin-induced transcriptional responses that are controlled by rapid turnover of unstable DNA-binding factors. Previous studies in our laboratory [32] and the data presented in this work show that *PID* transcript levels increase in both Dex-treated CK2mut plants and TBB-treated wild-type plants, indicating that up-regulation of *PID* occurs rapidly by inhibition of CK2 activity and endures for as long as we keep the CK2 inhibitor.

As other authors previously reported [39], here we show that *PID* transcription was up-regulated by auxin; however, its two closest homologues, *WAG1* and *WAG2*, were differentially regulated under the conditions used in this work, as their transcript levels either remained steady or decreased, respectively. We also show that *PID* upregulation by auxin was impaired in TBB-treated plants only when TBB was simultaneously present with exogenous auxin, and that, as soon as TBB was removed, NAA could resume *PID* transcriptional activation. These results support the idea that CK2 activity is necessary in the initial steps of the auxin-signaling pathway. Auxin-induced gene transcription is mainly controlled by a family of proteins called auxin repressors (the AUX/ IAA protein family) that dimerize with auxin response factors (ARFs) bound to auxin response elements, repressing early/primary auxin responses. The AUX/IAA repressors are short-lived proteins and their stability and activity are modulated by auxin, which promotes their ubiquitination and degradation via the 26S proteosome. As a consequence, inhibition of protein synthesis *in vivo* should potentiate the accumulation of auxin-regulated gene transcripts, due to depletion of protein repressors available inside the cell [35]. We show here that incubation with cycloheximide, an inhibitor of protein synthesis, resulted in high increase of *PID* transcript levels, supporting the idea that the short-lived repressors regulate *PID* expression. Interestingly, the cycloheximide effect was partially reversed in the presence of TBB, suggesting that inhibition of CK2 might increase the stability of the AUX/IAA repressors. This hypothesis was tested by using Arabidopsis plants containing a translational fusion of AXR3 (an AUX/IAA repressor) with GUS. Our results demonstrated that inhibition of CK2 resulted in stabilization of AXR3, and that this effect could not be reversed by exogenous auxin. These data are in agreement with a role for CK2 as a positive regulator of auxin-signaling pathways, its activity being required for degradation of auxin repressors. We do not know yet whether the effect is produced by the high SA content of CK2-defective plants, or CK2 activity is directly regulating either AXR3 post-translational modification (and its subsequent targeting for degradation) and /or proteosome activity.

The importance of the SA pathway in the up-regulation of *PID* transcription was disclosed by using the SA-defective *NahG* Arabidopsis mutant, as TBB-induced accumulation of *PID* transcripts was impaired in the *NahG* background. Moreover, our results show that auxin- and SA-pathways are tightly connected in the regulation of *PID* transcription and they act antagonistically. Interestingly, several components of the SA-signaling pathway, such as NPR1 (a master regulator of SA-signaling), are targeted for degradation via the 26S-proteosome in a SA-dependent manner [49,50]. In this scenario, proteosome inhibition by MG132, whereas inhibiting auxin-signaling by blocking degradation of auxin repressors, might activate SA-signaling by increasing the NPR1 half-life. Furthermore, NPR1 activity as a co-transcription factor depends on SA accumulation [51–53]. We can speculate that this double and antagonistic role of SA in controlling NPR1 stability and activity might explain the lower accumulation of *PID* transcript levels in MG132-treated *NahG* plants as compared to MG132-treated wild-type plants.

**Fig 5:**
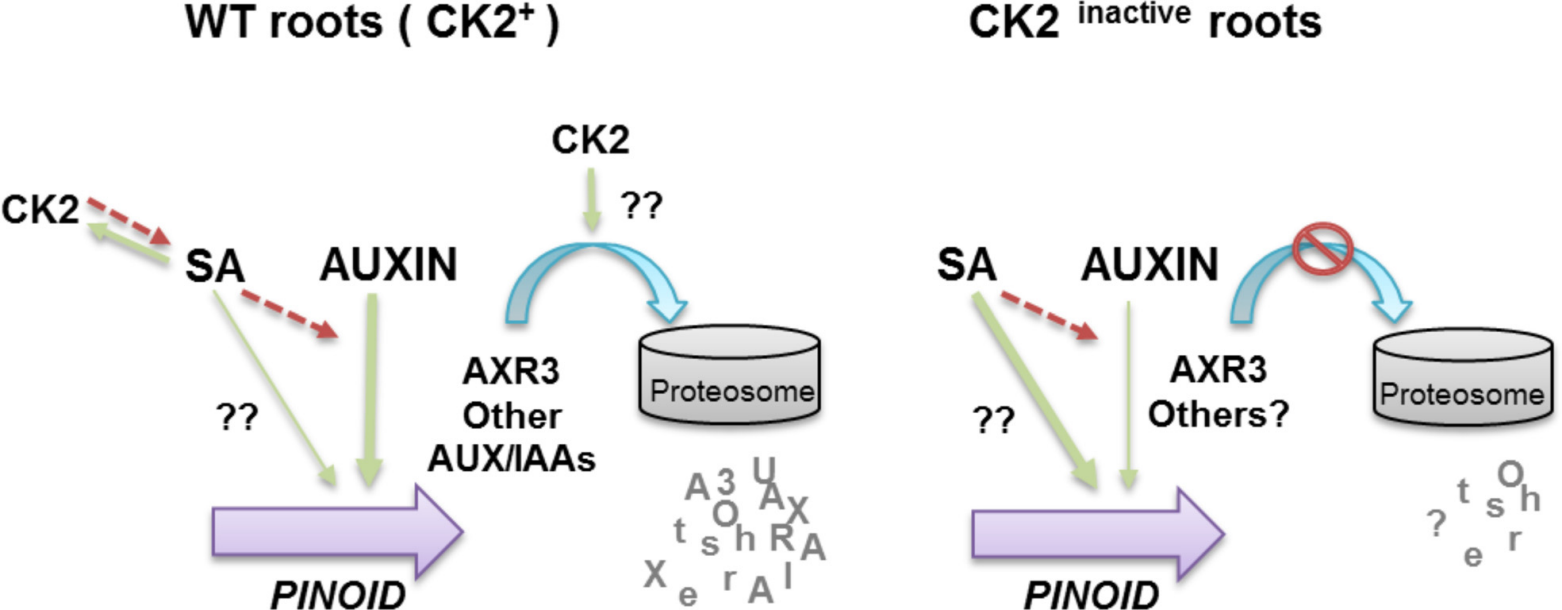
Proposed model for the interplay between auxin, salicylic acid and protein kinase CK2 in the regulation of *PINOID* transcription. Left panel: we propose that both auxin and SA are positive signals regulating *PINOID* (*PID*) transcription, and that CK2 activity is required in both pathways. The model shows the feed-back regulation between CK2 and SA previously reported (Plant J. 2014;78: 411–23. doi:10.1111/tpj.12481), and the role of CK2 in the regulation of auxin repressors stability (this work). Right panel: the model shows that under conditions of CK2 depletion the auxin-signaling pathway is repressed by stabilization of the AXR3 repressor (and might be of other members of the AUX/IAA family), whereas SA-signaling is activated due to increased SA levels. Green arrow, positive regulation; red discontinuous arrow, negative regulation.

Taken together, our results show that both auxin and SA are positive signals controlling *PID* transcript accumulation. It has been demonstrated by other authors that a negative feed-back between SA and auxin-signaling is exploited in plant defense mechanisms [28] and, here, we show that this cross-talk is also operating in the regulation of *PID* transcription. In addition, we show that protein kinase CK2 is an important player of this loop, as it regulates SA homeostasis [29] and auxin repressors stability (this work). Our proposed model (Fig 5) shows the two key points in which CK2 activity might modulate *PID* expression (left panel). Consequently, under conditions of CK2 inhibition (right panel), the auxin-signaling pathway is repressed and the SA-signaling activated (due to increased SA levels). Moreover, we cannot discard a more general role of CK2 as a regulator of protein degradation via the 26S proteosome. In that case, CK2 activity would affect almost every signaling pathway within eukaryotic cells.

## Supporting Information

S1 Fig *WAG1* and *WAG2* transcriptional responses to auxin in wild-type and CK2mut backgrounds. Transcript levels were measured in roots and normalized to those of *EF-1-α* gene. Data were expressed as fold changes of gene expression relative to the levels measured in control plants, and represented in logarithmic scale (log_10_). Graphs show the mean of three biological replicates ± standard deviation. Same letters above the bars indicate no significant differences from each other (ANOVA P≤0.05). Dex, dexamethasone; NAA, 1-naphthaleneacetic acid; 2,4-D, 2,4 dichlorophenoxyacetic acid.
(TIF)

S2 Fig Transcriptional regulation of auxin transporters. Gene transcript levels were measured in roots and are shown as relative expression to *EF-1-α* transcript levels. Error bars indicate standard deviations. Same letters above the bars indicate not significant differences from each other (ANOVA P≤0.05). Abbreviations: Dex, dexamethasone; NAA, 1-naphthaleneacetic acid; 2,4-D, 2,4-dichlorophenoxyacetic acid.
(TIF)

S3 Fig Interplay between protein kinase CK2 and protein synthesis in the transcriptional regulation of *PIN* genes. Gene transcript levels were measured in roots and normalized to those of *EF-1-α* gene. Data are expressed as fold changes of gene expression relative to the levels measured in control plants. Graphs show the mean of two biological replicates ± standard deviation. Asterisks mean significant differences (P≤0.05, Student’s t-test). Abbreviations: TBB, 4,5,6,7-tetrabromo benzotriazol; CHX, cycloheximide.
(TIF)
