# Supplementary figures and images for: The Protein Kinase CK2 Mediates Cross-Talk between Auxin- and Salicylic Acid-Signaling Pathways in the Regulation of PINOID Transcription
Source: PLoS One. 2016 Jun 8;11(6):e0157168. doi: 10.1371/journal.pone.0157168 (PMC4898841; doi:10.1371/journal.pone.0157168)

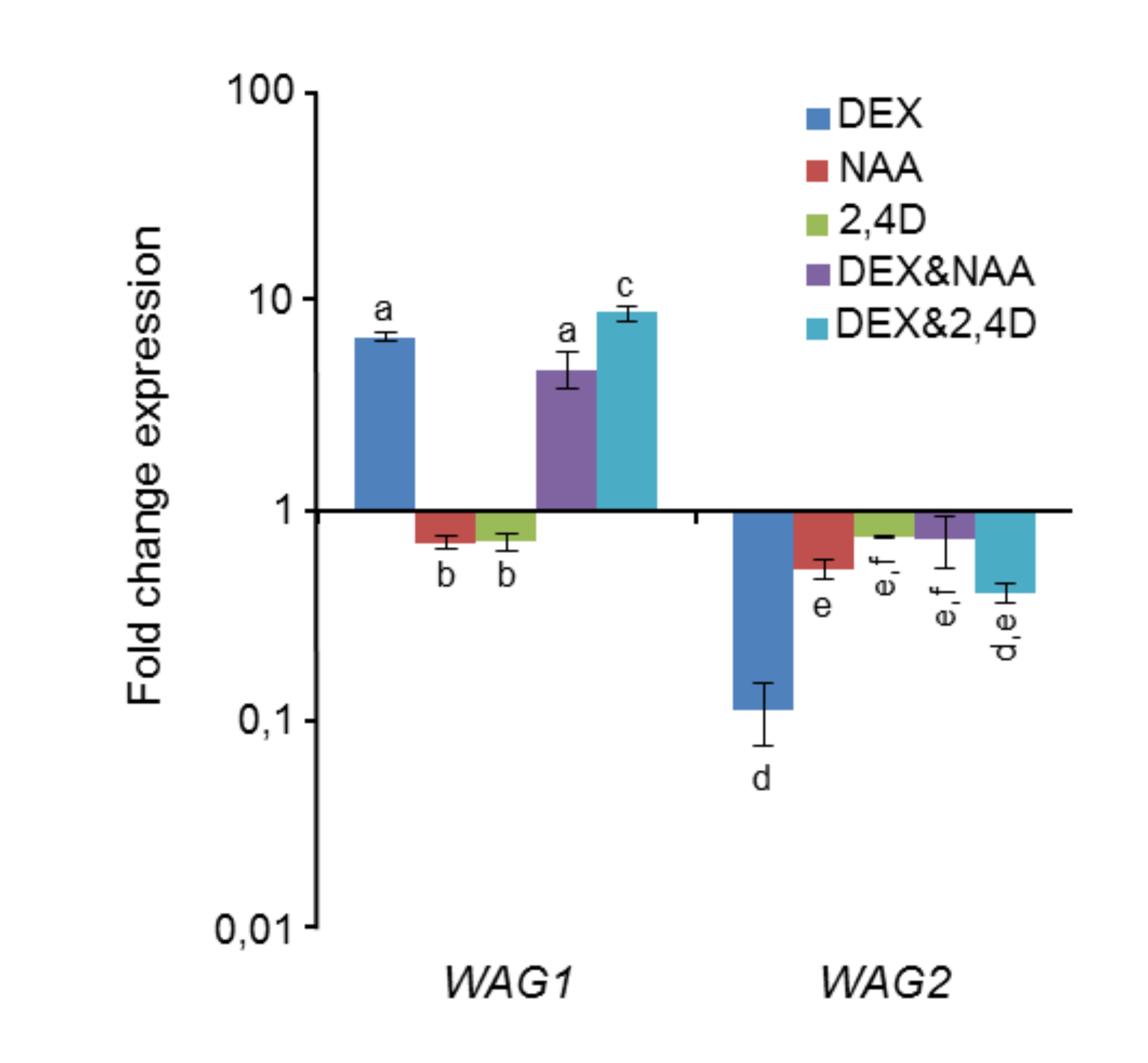

Supplement: S1 Fig — Transcript levels were measured in roots and normalized to those of EF-1-α gene. Data were expressed as fold changes of gene expression relative to the levels measured in control plants, and represented in logarithmic scale (log10). Graphs show the mean of three biological replicates ± standard deviation. Same letters above the bars indicate no significant differences from each other (ANOVA P≤0.05). Dex, dexamethasone; NAA, 1-naphthaleneacetic acid; 2,4-D, 2,4 dichlorophenoxyacetic acid. (TIF) [file pone.0157168.s001.TIF]

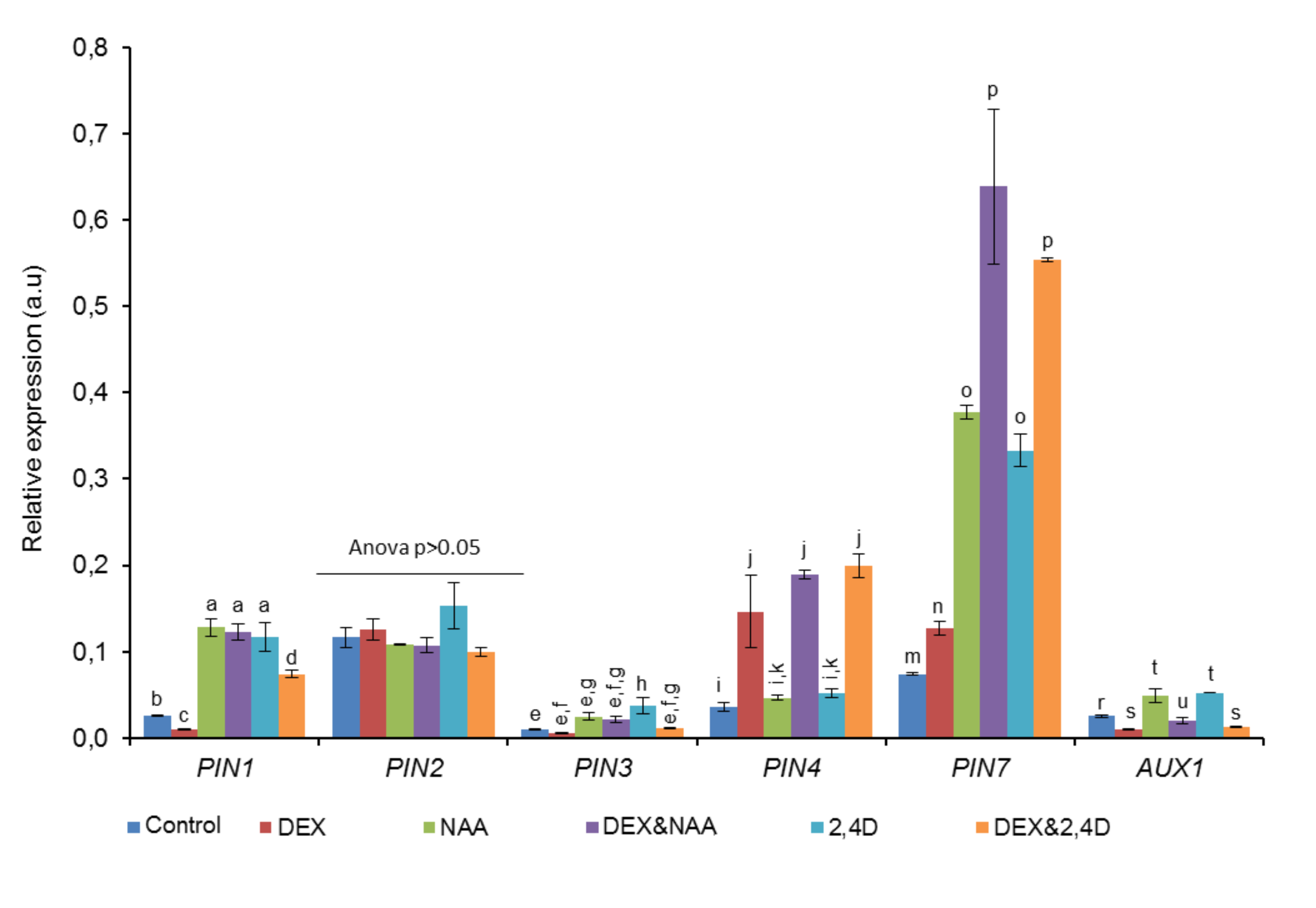

Supplement: S2 Fig — Gene transcript levels were measured in roots and are shown as relative expression to EF-1-α transcript levels. Error bars indicate standard deviations. Same letters above the bars indicate not significant differences from each other (ANOVA P≤0.05). Abbreviations: Dex, dexamethasone; NAA, 1-naphthaleneacetic acid; 2,4-D, 2,4-dichlorophenoxyacetic acid. (TIF) [file pone.0157168.s002.tif]

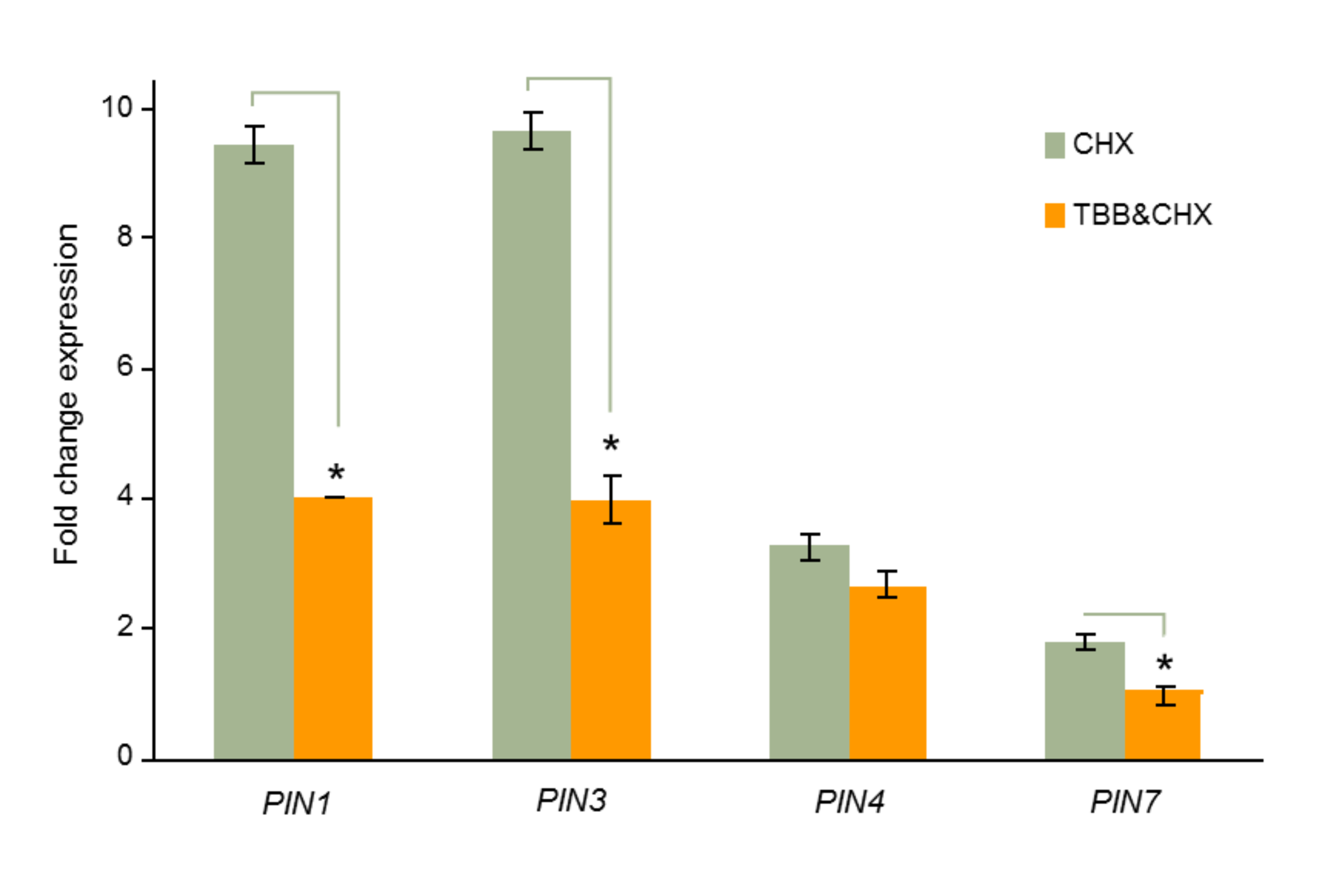

Supplement: S3 Fig — Gene transcript levels were measured in roots and normalized to those of EF-1-α gene. Data are expressed as fold changes of gene expression relative to the levels measured in control plants. Graphs show the mean of two biological replicates ± standard deviation. Asterisks mean significant differences (P≤0.05, Student’s t-test). Abbreviations: TBB, 4,5,6,7-tetrabromo benzotriazol; CHX, cycloheximide. (TIF) [file pone.0157168.s003.tif]
